# Lithiophilic Modification of Self-Supporting Carbon-Based Hosts and Lithium Metal Plating/Stripping Behaviors

**DOI:** 10.3390/nano15100746

**Published:** 2025-05-15

**Authors:** Zipeng Jiang, Shoudong Xie, Guijun Yang, Huiyuan Chen, Jiahang Lv, Ang Li, Chengwei Fan, Huaihe Song

**Affiliations:** 1 Qinghai Provincial Key Laboratory of Advanced Materials and Applied Technology, Qinghai University, Xining 810016, China; zpjiang@qhu.edu.cn (Z.J.);; 2 College of Chemical Engineering, Qinghai University, Xining 810016, China; 3 State Key Laboratory of Chemical Resource Engineering, Beijing Key Laboratory of Electrochemical Process and Technology for Materials, Beijing University of Chemical Technology, Beijing 100029, China; 4 College of Chemistry and Environmental Engineering, Xinjiang Institute of Engineering, Urumuqi 830029, China

**Keywords:** lithium metal anode, lithiophilic carbon-based host, corrosions, plating/stripping behaviors

## Abstract

Metallic lithium anodes possess the lowest redox potential (−3.04 V vs. SHE) and an ultra-high theoretical capacity (3860 mAh g^−1^, 2061 mAh cm^−3^). However, during electrochemical cycling, lithium metal tends to plate unevenly, leading to the formation of lithium dendrites. Moreover, severe electrochemical corrosion occurs at the interface between metallic lithium and traditional copper foil current collectors. To address these issues, we selected corrosion-resistant carbon paper as a lithium metal host and modified a uniform distribution of silver nanoparticles and a F-doped amorphous carbon structure as a highly lithiophilic F-CP@Ag host to enhance lithium-ion transport kinetics and achieve improved affinity with lithium metal. The silver nanoparticles reduced the lithium nucleation energy barrier, while F doping resulted in a LiF-rich solid electrolyte interphase that better accommodated volume changes in lithium metal. These two strategies worked together to ensure uniform and stable lithium metal plating/stripping on the F-CP@Ag host. Consequently, under the conditions of 1 mA cm^−2^ and 1 mAh cm^−2^, the symmetric cell exhibited stable cycling with a polarization voltage of 8 mV for up to 1400 h. This work highlights the corrosion problem of lithium metal on traditional copper foil current collectors and provides guidance for the long-term cycling stability of lithium metal anodes.

## 1. Introduction

Metallic lithium, with the lowest redox potential (−3.04 V vs. SHE) and an ultra-high theoretical capacity (3860 mAh g^−1^, 2061 mAh cm^−3^), is regarded as a promising anode material for next-generation high-energy-density lithium secondary batteries. However, the chemical environment surrounding the current collector in lithium-ion batteries (LIBs) differs from that in lithium metal batteries (LMBs). In conventional LIBs, a dense active material layer is coated onto the current collector, forming a stable electrode structure. In contrast, the significant volume fluctuation of lithium metal during cycling compromises the mechanical integrity of the active material, leading to a loose and porous morphology in the current collector [1]. As a result, the current collector is often directly exposed to the electrolyte with metallic lithium, making it susceptible to electrochemical corrosion [2].

**Figure 1:**
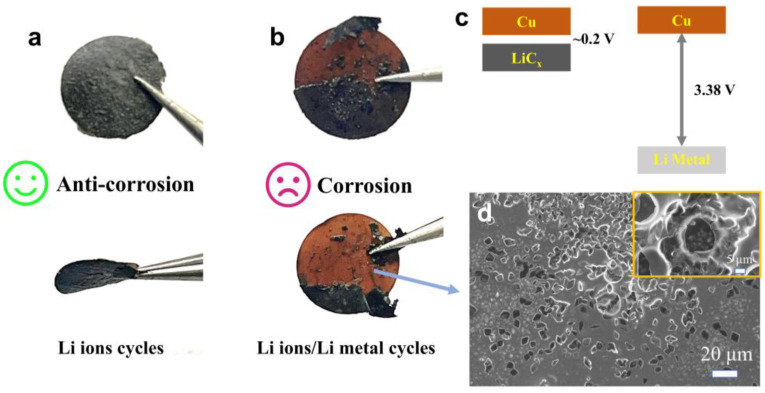
Surface morphology of the copper foil current collector after electrochemical corrosion induced by lithium-ion (**a**) and lithium metal plating (**b**,**c**). SEM image of copper foil surface after Li ions/Li metal cycles (**d**).

As shown in Figure 1, carbon-coated copper foil electrodes subjected to lithium-ion and lithium metal cycling protocols exhibit markedly different surface morphologies. The electrode remains relatively intact after cycling in the lithium-ion system, whereas in the lithium metal system, severe delamination of the active material from the current collector occurs due to lithium plating/stripping. Furthermore, as illustrated in Figure 1c, galvanic corrosion occurs when two dissimilar metals are electrically connected and immersed in a corrosive electrolyte. In this process, electrons transfer from the metal with the lower electrochemical potential (anode) to the metal with the higher potential (cathode). Specifically, lithium metal (E^θ^_Li|Li⁺_ = −3.04 V vs. SHE) acts as the anode, undergoing spontaneous oxidation and dissolution, while copper foil (E^θ^_Cu|Cu_^2^⁺ = +0.34 V vs. SHE) serves as the cathode, where reduction reactions occur. The huge potential difference (3.38 V) between lithium and copper significantly accelerates electrochemical corrosion at their interface [3]. As shown in Figure 1d, severe corrosion pits can be observed on the copper foil surface, a process that promotes the formation of electrically isolated “dead lithium”, leading to continuous electrolyte depletion and capacity fading [4].

Carbon-based materials, as traditional inorganic conductors, possess high electronic conductivity, a tunable structural morphology, and excellent mechanical stability [5]. Unlike copper foil, carbon materials are resistant to galvanic corrosion in lithium metal batteries. However, their inherent poor wettability toward lithium increases the nucleation energy barrier for lithium plating on heterogeneous surfaces [6,7]. Our previous studies [8,9] demonstrated that amorphization treatments and lithiophilic element modification are effective strategies to improve the lithium affinity of carbon-based current collectors, thereby enhancing lithium plating/stripping performance. Zuo et al. [10] directly employed graphitized carbon fibers as three-dimensional current collectors to achieve dendrite-free lithium plating. Yang et al. [11] synthesized ultra-fine silver nanoparticles (~40 nm) on nanocarbon fibers via rapid Joule heat. Zhang et al. [12] fabricated a coral-like silver-plated carbon fiber composite lithium anode via electroplating followed by molten lithium infusion, effectively reducing the lithium plating overpotential. Li et al. [13] pre-coated silver nanoparticles onto carbon fiber paper by the silver mirror reaction, which lowered the lithium nucleation overpotential. However, achieving a uniform dispersion of silver nanoparticles remains challenging, and the synthesis process is relatively complex.

Moreover, numerous studies have demonstrated that LiF-rich solid electrolyte interphase (SEI) layers facilitate stable lithium plating and promote uniform lithium plating. As a result, significant research attention has been directed toward fabricating LiF-enriched SEI layers. Apart from electrolyte additives, C-F bonds in fluorine-doped carbon materials can induce the formation of LiF-rich SEI [14]. Since fluorine and other heteroatoms primarily incorporate into defect sites (vacancies or edges) within graphite lattices, the doping efficiency is often limited by the inert nature of conventional carbon surfaces. Therefore, introducing vacancies or edge sites in graphite lattices is crucial for achieving efficient fluorine doping in carbon materials [15].

Herein, we utilized AgNO_3_ as the silver precursor and PVDF as the fluorine precursor, dispersing them homogeneously in N-methylpyrrolidone (NMP) solution, followed by blade-coating onto artificial carbon paper (CP). After carbonization, a fluorine-doped, highly lithiophilic carbon material uniformly loaded with silver nanoparticles (F-CP@Ag) was successfully synthesized. This study investigates the carbon-based material’s potential as a lithium metal host through material characterization and electrochemical analysis, elucidating the relationship between the structural properties of the material and lithium-ion transport kinetics, as well as the correlation between fluorine doping and SEI formation. 

## 2. Materials and Methods

### 2.1. Synthesis of Carbon-Based Host Materials

First, 100 mg of AgNO_3_ and 300 mg of PVDF were dissolved in 10 mL of NMP solution under magnetic stirring for 12 h to form a stable solution. Meanwhile, commercial CP was pre-cleaned by sequential immersion in 1 M HCl and deionized water. After drying, the cleaned CP was placed on a clean glass substrate, and the prepared solution was drop-cast onto the CP, followed by uniform blade coating. The coated samples were then dried in a vacuum oven at 80 °C for 12 h to remove residual solvent. Subsequently, the dried samples were carbonized in a tube furnace by heating at a ramp rate of 5 °C min^−1^ to 500 °C, followed by an isothermal holding period of 3 h.

As a result, a fluorine-doped, lithiophilic carbon material uniformly loaded with silver nanoparticles (F-CP@Ag) was successfully obtained on the CP. Finally, the carbonized CP was punched into circular electrode discs (12 mm in diameter).

### 2.2. Analysis and Measures

To examine the morphology, size distribution, and microstructure of the carbon paper samples and their corresponding electrodes, we utilized scanning electron microscopy (SEM; ZEISS SUPRA 55; Oberkochen, Germany), transmission electron microscopy (TEM; Hitachi 7700; Puchong, Malaysia), and high-resolution transmission electron microscopy (HRTEM; Talos F200X G2; Thermo Scientific, Waltham, MA, USA). Crystallographic analysis of the carbon and silver phases in the samples was performed through X-ray diffraction (XRD; Philips Rigaku D/max 2500B2+/PCX; Tokyo, Japan) using filtered Cu Kα radiation (λ = 1.54056 Å). The elemental composition and chemical states of carbon paper samples and electrodes were investigated via X-ray photoelectron spectroscopy (XPS; Thermo VG ESCALAB 250; Waltham, MA, USA, utilizing Al Kα radiation as the excitation source). Additionally, confocal Raman spectroscopy (Raman, Renishaw in Via-Reflex; Aramis, Jobin Yvon, Arcueil, France), employing 532 nm laser excitation, was conducted to probe structural defects within the sample.

### 2.3. Electrochemical Measurements

Electrochemical performance was evaluated using CR2032-type coin cells (Guangdong Canrd New Energy Technology Co., Ltd., Dongguan, China), which were assembled in an argon-filled glovebox with O_2_ and H_2_O levels maintained below 0.1 ppm. The electrolyte consisted of 1 M lithium bis(trifluoromethanesulfonyl)imide (LiTFSI) dissolved in a 1:1 (*v*/*v*) mixture of 1,3-dioxolane (DOL) and 1,2-dimethoxyethane (DME), with 2 wt% LiNO_3_ as an additive to enhance interfacial stability. For half-cells, lithium metal foil was employed as the counter electrode, while CP and F-CP@Ag served as the working electrodes, each with an average diameter of 10 mm for lithium plating. For symmetric cells, a fixed areal capacity of 4 mA h cm^−2^ of lithium metal was electrochemically plated onto the CP and F-CP@Ag electrodes. The pre-plated electrodes were subsequently disassembled and reassembled into symmetric cells to evaluate their long-term electrochemical stability. Lithium plating was performed at current densities ranging from 0.5 to 3.0 mA cm^−2^, with an areal capacity controlled between 1 and 2 mA h cm^−2^, followed by lithium stripping up to 1.0 V at the same rate. All cells were pre-cycled for 5 cycles between 0.01 and 1 V at 0.5 mA cm^−2^ to stabilize the SEI before further testing. Galvanostatic cycling was conducted using a Land CT3001A battery tester (Wuhan, China) at 25 °C. Electrochemical impedance spectroscopy (EIS) was performed using a CHI760E electrochemical workstation (Shanghai, China), covering a frequency range of 100 kHz to 0.01 Hz, with an AC perturbation amplitude of 5 mV.

## 3. Results

### 3.1. Structural Characteristics of the Carbon-Based Host

**Figure 2:**
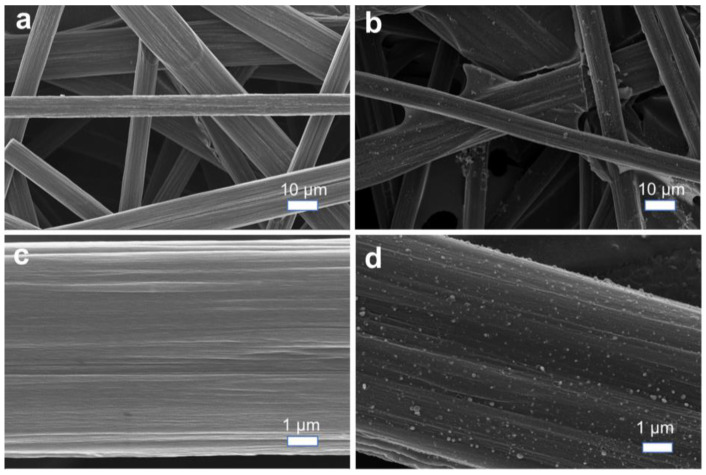
SEM images of CP (**a**,**c**) and F-CP@Ag (**b**,**d**).

In this study, we selected commercial artificial CP with a high degree of graphitization as the lithium metal host and performed lithiophilic modifications. Initially, we employed a simple silver ammonia solution reduction method to load silver nanoparticles onto the CP surface. SEM characterization revealed that, while silver nanoparticles were successfully deposited onto the smooth CP surface, severe agglomeration occurred. Subsequent electrochemical testing demonstrated that the nonuniform silver nanoparticle distribution exacerbated lithium dendrite formation and the subsequent generation of dead lithium, resulting in unstable electrochemical cycling. To address this issue, we adopted an alternative approach using AgNO_3_ as the silver source and PVDF as the fluorine source, dispersing them in an organic NMP solvent with a lower polarity. After carbonization, we successfully fabricated a fluorine-doped lithiophilic carbon material with uniformly distributed silver nanoparticles (F-CP@Ag) on the CP substrate. As shown in Figure 2a,b, the pristine CP consists of interwoven smooth carbon fibers forming a three-dimensional porous structure, with fiber diameters of approximately 10 μm. The F-CP@Ag material exhibits homogeneously dispersed silver nanoparticles that are firmly anchored on the fiber surfaces. Furthermore, these fibers themselves become roughened after modification (Figure 2c,d).

**Figure 3:**
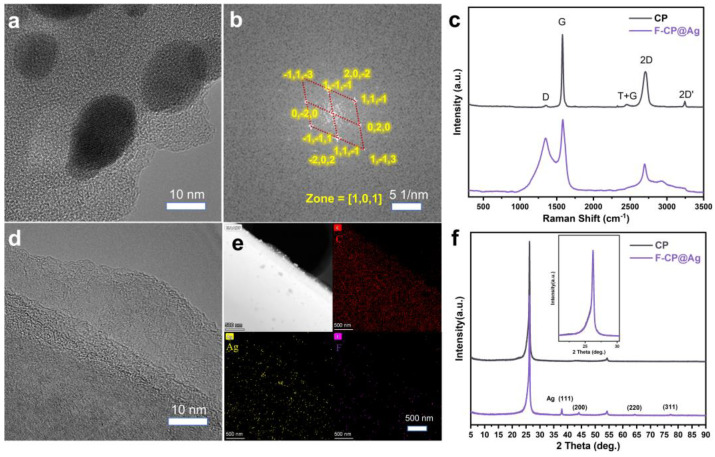
HRTEM images of F-CP@Ag (**a**), crystalline structure of Ag (**b**), elemental mapping (**e**), and CP (**d**). Raman spectra (**c**) and XRD patterns (**f**) of samples.

TEM analysis confirmed that both pristine and modified CP materials retained a densely packed fibrous structure. The unmodified CP exhibits an orderly crystalline structure characteristic of graphitic hexagonal rings (Figure 3d). In contrast, the silver nanoparticles in F-CP@Ag are encapsulated within a highly disordered amorphous carbon layer (Figure 3a) generated via precursor pyrolysis. Previous studies have shown that low-crystallinity, defect-rich carbon layers facilitate lithium-ion diffusion and promote uniform lithium plating. Additionally, encapsulating silver nanoparticles within the carbon layer prevents their detachment from the material surface during cycling, thereby enhancing structural stability. The silver nanoparticles, approximately 10 nm in diameter, exhibit high crystallinity, which facilitates rapid Li-Ag alloy formation, thereby enhancing the lithiophilic properties of the material. The Fourier transform of the red-marked region in Figure 3a yields a diffraction pattern characteristic of a single-crystal structure (Figure 3b) when compared with the body-centered cubic (BCC) Ag lattice (PDF#01-1164), confirming that the deposited silver nanoparticles possess a highly crystalline BCC structure. Elemental mapping further demonstrates the homogeneous distribution of Ag and F throughout the material (Figure 3e).

Raman spectroscopy, a powerful tool for characterizing carbon structures, was used to analyze the materials’ defect characteristics [16]. The G band indicates the typical degree of graphitization, while the D band corresponds to defect-induced finite crystallite size effects. A high-intensity symmetric 2D band signifies a well-ordered graphitic structure [17]. The CP sample exhibits strong G and 2D bands with a weak D band, confirming its highly graphitized characteristics. In contrast, F-CP@Ag displays convolution peaks in the 1000 cm^−1^ to 2000 cm^−1^ range, indicative of abundant surface defects (Figure 3c).

Given that carbon materials exhibit relatively low X-ray absorption coefficients, XRD analysis was employed to probe for deeper structural information [16]. Both the CP and F-CP@Ag materials exhibit sharp (002) diffraction peaks, indicating the presence of well-ordered three-dimensional (3D) stacked carbon layers. Additionally, the (004) peak near 54° further confirms the coexistence of graphitic stacking and turbostratic structures within the materials [18]. After normalizing the diffraction peak intensities, we found that both the (002) peak positions and shapes of the CP and F-CP@Ag samples are nearly identical. Notably, F-CP@Ag exhibits distinct diffraction peaks corresponding to Ag (111) and other major crystal planes (Figure 3f), suggesting that the modification primarily affects the surface of the materials’ micro- and nanostructural characteristics.

**Figure 4:**
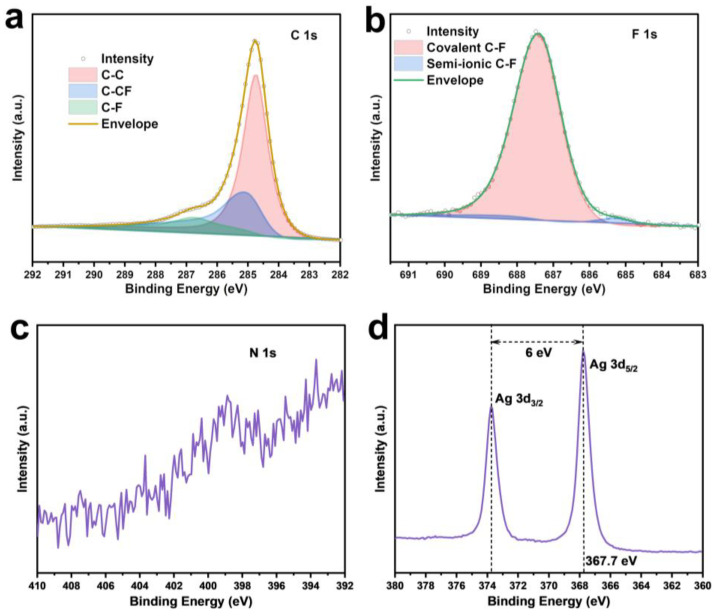
XPS fine spectra of F-CP@Ag sample: C1s (**a**), F1s (**b**), N1s (**c**), Ag3d (**d**).

XPS analysis was conducted to investigate the surface elemental composition and chemical states of F-CP@Ag. The F and Ag contents were determined to be 3.2 at% and 5.1 at%, respectively. The high-resolution C1s spectrum (Figure 4a) exhibited peaks at 284.8, 286.1, and 288.4 eV, corresponding to sp^2^ C-C, sp^2^ C-CF, and sp^3^ C-F bonds, respectively [19,20,21]. The F1s spectrum (Figure 4b) featured a main peak at 687.4 eV and a minor peak at 685.7 eV, which can be attributed to covalent and free-state C-F bonding, respectively, consistent with the C1s spectral fitting. Fluorine doping facilitates the formation of a LiF-rich SEI layer on the electrode surface. The weak N1s peak (Figure 4c) indicated that NMP solvent-induced N doping in CP is negligible. The binding energy of Ag3d_5_/_2_ exhibits a 0.6 eV shift to a lower energy compared to standard metallic Ag (368.2 eV), suggesting nanoscale silver features and partial Ag-C bonding [22]. Furthermore, the Ag3d region displays well-separated spin–orbit components (Δmetal = 6.0 eV), with asymmetric Ag3d_5_/_2_ and Ag3d_3_/_2_ peaks, confirming that silver remained predominantly in the metallic state (Figure 4d).

### 3.2. Electrochemical Performance of Lithium Plating/Stripping

**Figure 5:**
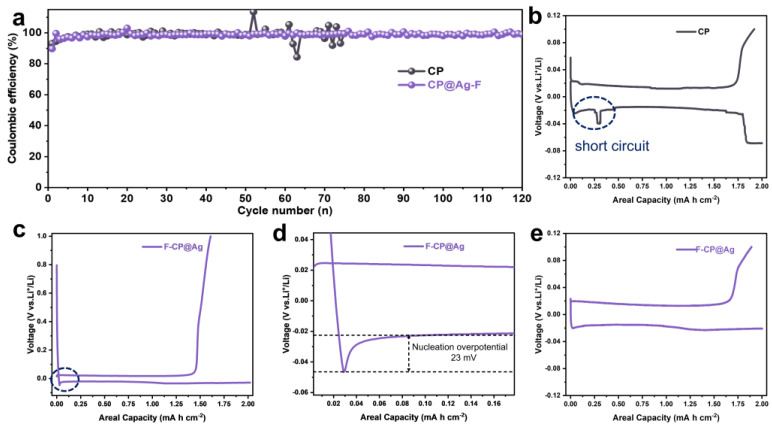
Cycling performance of lithium plating/stripping (0.5 mA cm^−2^, 2 mAh cm^−2^) in half-cells (**a**). Voltage profiles of CP (**b**) and F-CP@Ag (**c**) and a partial enlarged view (**d**) in 5th cycles. Voltage profiles of F-CP@Ag (**e**) in 100th cycles.

Half-cells were assembled using either F-CP@Ag or CP as the working electrode with lithium foil as the counter electrode to evaluate the electrochemical performance of lithium plating/stripping on the carbon-based host. As shown in Figure 5a, pristine CP exhibits significant Coulombic efficiency fluctuations after 50 cycles due to the lithiophobic interfacial layer, leading to uncontrolled lithium dendrite growth and SEI rupture, which results in inevitable lithium and electrolyte consumption. In contrast, F-CP@Ag half-cells exhibit significantly enhanced cycle life while maintaining a high Coulombic efficiency. This improvement may be attributed to the uniform Ag lithiophilic sites and F doping-induced LiF-rich SEI formation, which guide uniform lithium plating.

Comparison of the initial lithium plating/stripping capacity–voltage profiles revealed that CP half-cells exhibit transient soft short circuits [23,24] and severe voltage drops before reaching the designated lithium plating capacity (Figure 5b). In contrast, F-CP@Ag displays stable lithium plating/stripping characteristics (Figure 5c). A magnified view of the dashed region in Figure 5c revealed the nucleation overpotential of lithium at only 23 mV (Figure 5d), indicating a significantly reduced nucleation energy barrier, which facilitates uniform metallic lithium plating. Over 100 cycles, highly stable lithium plating/stripping behavior was observed (Figure 5e).

**Figure 6:**
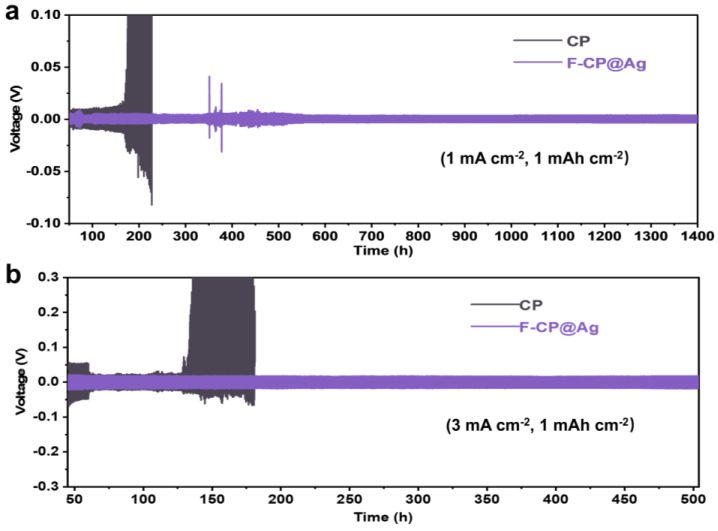
Cyclic performance of symmetric cells: (**a**) (1 mA cm^−2^, 1 mAh cm^−2^), (**b**) (3 mA cm^−2^, 1 mAh cm^−2^).

To evaluate the interfacial stability of lithium metal during long-term cycling, electrodes plated with a controlled amount of lithium were assembled into symmetric cells (Figure 6). The F-CP@Ag symmetric cell exhibits stable cycling for over 1400 h at a relatively low current density of 1 mA cm^−2^, with a consistently low hysteresis voltage of approximately 8 mV, despite some voltage fluctuations attributed to intermittent soft short circuits. In contrast, the CP symmetric cell exhibits a sharp increase in hysteresis voltage after 160 cycles, suggesting that the fracture of lithium dendrites leads to the formation of a significant amount of “dead lithium”, increasing internal resistance and polarization. Even under more demanding conditions (3 mA cm^−2^), the F-CP@Ag symmetric cell maintains stable cycling for over 500 h, with a steady hysteresis voltage of 38 mV. Conversely, the CP symmetric cell undergoes soft short circuits after only 60 cycles, where lithium dendrites penetrate the separator, causing a sudden voltage drop. In subsequent cycles, fractured lithium dendrites allow continued plating/stripping for a limited period, but the accumulation of “dead lithium” results in a rapid increase in internal resistance, ultimately leading to complete failure after approximately 100 cycles.

Therefore, compared to the CP host, the F-CP@Ag electrode demonstrates significantly improved lithium plating/stripping cycling stability in both half-cell and symmetric cell configurations. This enhancement is likely attributed to the lithiophilic Ag nucleation sites and fluorine doping, which facilitate the formation of a more robust and stable interfacial environment through favorable interactions with the electrolyte.

### 3.3. Mechanistic Analysis of Lithium Plating/Stripping Behavior

**Figure 7:**
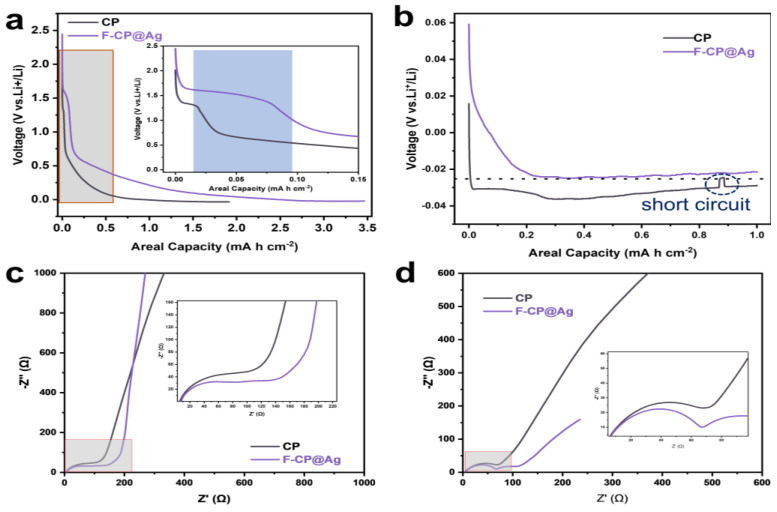
Capacity–voltage curves of lithium plating: (**a**) 1st cycle, (**b**) 2nd cycle. EIS of electrodes (**c**) in 1st and (**d**) after 5th cycles.

To achieve deeper insights into the electrochemical differences between the different hosts, the lithium affinity of the substrates was evaluated based on the voltage profiles of the first and second plating/stripping cycles. As shown in Figure 7a, the F-CP@Ag electrode exhibits a significantly higher initial discharge capacity (3.47 mAh cm^−2^) compared to CP (1.92 mAh cm^−2^) when discharged to 0 V. This enhancement can be attributed to the alloying reaction between Ag and Li, which contributes additional capacity, as well as the amorphous carbon layer, which provides a sloped capacity region. The inset of Figure 7a highlights distinct differences in SEI formation behavior between the two electrodes. During SEI formation at approximately 1.5 V, the F-CP@Ag electrode exhibits a slightly elevated potential compared to CP. Notably, the SEI formation capacity on CP is merely 0.01 mAh cm^−2^, whereas F-CP@Ag demonstrates a significantly higher value of 0.07 mAh cm^−2^. This discrepancy is likely attributable to the transformation of C-F bonds into a LiF-rich SEI, which consumes additional lithium; this phenomenon will be discussed in detail later.

The second-cycle plating profiles (Figure 7b) reveal that the F-CP@Ag electrode possesses a substantially lower nucleation overpotential, with a nucleation potential of approximately −23 mV, in contrast to CP, which displays a higher nucleation potential of −35 mV. Moreover, CP exhibits multiple unstable plateaus and abrupt voltage fluctuations in the high-capacity region, indicating nonuniform lithium plating that leads to irregular polarization variations. These findings collectively suggest that the intrinsic lithiophobic characteristics of the CP surface are unfavorable for uniform lithium nucleation and growth. In contrast, the synergistic effects of surface fluorination and the introduction of lithiophilic silver significantly enhance lithium plating homogeneity. Specifically, the fluorine-enriched surface facilitates the formation of a LiF-rich SEI, thereby improving interfacial stability. Concurrently, the homogeneously dispersed Ag nanoparticles are alloyed with lithium, providing abundant nucleation sites that reduce the nucleation energy barrier and promote uniform lithium plating.

EIS was employed to further investigate SEI characteristics and lithium-ion transport properties. The middle- to low-frequency region of the Nyquist plot corresponds to the lithium-ion diffusion capability, where a steeper slope indicates higher lithium-ion mobility. As shown in Figure 7c, the F-CP@Ag electrode exhibits superior lithium-ion transport kinetics compared to the CP electrode, which facilitates rapid lithiation of Ag nanoparticles on the electrode surface, thereby enhancing lithium affinity. Additionally, lithium ions diffuse more efficiently into the carbon fiber matrix of the F-CP@Ag electrode, leading to a higher degree of lithiation within the 3D conductive network compared to the CP electrode. This gradient lithiation, extending from the electrode surface to its interior, promotes more uniform and dense lithium plating/stripping, thereby improving cycling stability.

The semicircles in the middle- and high-frequency regions of the EIS spectra correspond to charge transfer resistance and SEI resistance, respectively. Unlike conventional lithium-ion batteries, where impedance arises predominantly at the electrode–electrolyte interface, the present system also includes the impedance between deposited lithium and the substrate, as well as between lithium metal and the electrolyte. Notably, the impedance semicircle of the F-CP@Ag electrode is slightly larger than that of CP, consistent with first-cycle plating data, wherein the F-CP@Ag electrode exhibits greater lithium consumption due to the formation of a thicker SEI. After five plating/stripping cycles (Figure 7d), the lithium-ion diffusion rate in the F-CP@Ag electrode slightly decreases compared to the CP electrode, while the high-frequency semicircle diameter also reduces. This suggests that although lithium-ion transport slightly slows in the F-CP@Ag electrode, the SEI resistance decreases significantly. Given that the initial SEI formation on the F-CP@Ag electrode is more extensive than on the CP electrode, it is inferred that continuous cycling led to a denser, more robust SEI layer. In contrast, the inherently thinner SEI on the CP electrode is more susceptible to repeated rupture and reformation, which compromises its stability and contributes to performance degradation.

### 3.4. Morphological Analysis of Lithium Plating

**Figure 8:**
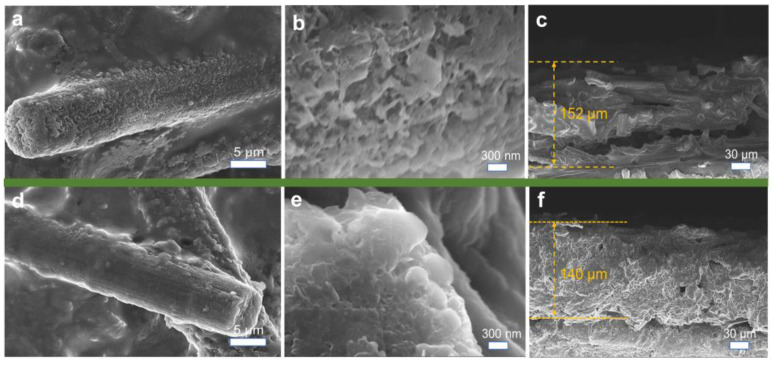
SEM images of the CP electrode’s (**a**,**b**) surface and (**c**) cross-section and the F-CP@Ag electrode’s (**d**,**e**) surface and (**f**) cross-section after plating/stripping cycles.

Ex situ SEM imaging was conducted to observe the surface morphology of the electrodes after lithium plating/stripping cycles (Figure 8). On a micron scale, the CP electrode exhibits numerous exposed voids and pores, whereas the F-CP@Ag electrode displays a denser surface morphology. As shown in Figure 8a, CP fibers are sparsely covered with loosely aggregated lithium fragments indicative of substantial “dead lithium” accumulation. In contrast, Figure 8d reveals that the F-CP@Ag fibers are coated with a uniform, spherical lithium layer, wherein lithium metal is evenly encapsulated within the SEI, with no observable large-scale dead lithium accumulation. At the nanoscale, needle-like lithium dendrites are distinctly visible on the CP electrode, which are prone to detachment from their roots during cycling, leading to dead lithium formation and deteriorated cycling stability. More critically, such dendritic structures pose a significant risk of separator penetration, potentially causing internal short circuits [25,26]. In contrast, the F-CP@Ag electrode exhibits a compact, block-like, or spherical lithium morphology, which is markedly different from the dendritic growth observed on the CP electrode. This uniform and compact lithium plating pattern is conducive to enhanced cycling stability and safety.

Cross-sectional analysis further corroborated these findings. After cycling, the CP electrode exhibits a thickness of 152 μm, with a loosely packed internal structure, wherein individual carbon fibers remain distinguishable, suggesting that lithium plating/stripping primarily occurs at the outermost layer. In contrast, the F-CP@Ag electrode exhibits a reduced thickness of 140 μm, characterized by a uniformly distributed and densely packed lithium layer throughout the electrode cross-section. This indicates that the entire 3D framework of the F-CP@Ag electrode actively participates in lithium plating/stripping. The introduction of surface fluorination and Ag nanoparticles significantly enhances the lithium affinity of the substrate, enabling full utilization of the 3D framework for mitigating volume expansion effects during cycling.

### 3.5. XPS Analysis of SEI Composition

**Figure 9:**
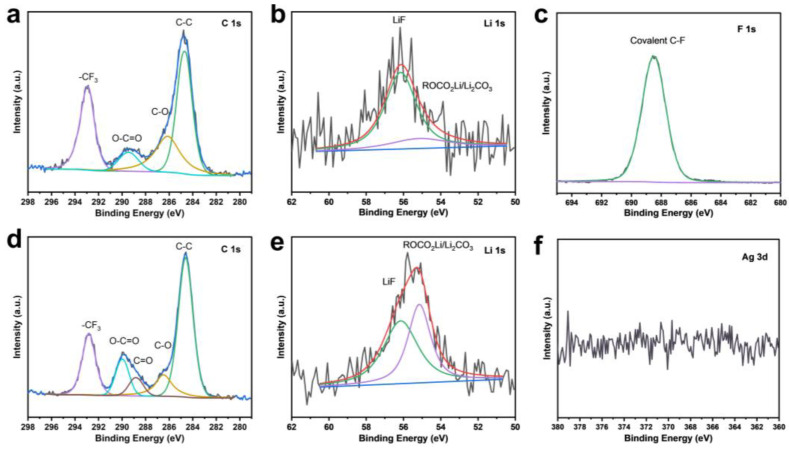
C_1s_ (**a**) and Li_1s_ (**b**) XPS fine spectra of CP and F_1s_ (**c**), C_1s_ (**d**), Li_1s_ (**e**) Ag_3d_ (**f**), and XPS fine spectra of F-CP@Ag after first cycle.

XPS analysis was performed after the first charge–discharge cycle to elucidate the elemental composition and chemical bonding states of the SEI (Figure 9). Comparative analysis of the C_1s_ spectra between the CP and F-CP@Ag electrodes revealed that the SEI formed on the F-CP@Ag electrode exhibits greater compositional complexity due to enhanced electrolyte decomposition. The Li_1s_ spectra of the CP electrode display a relatively weak signal intensity, indicative of a limited lithium content. Deconvolution of the spectra confirmed that the CP electrode primarily contains LiF structures, with relatively low proportions of organic and inorganic SEI components. In contrast, the Li_1s_ spectra of the F-CP@Ag electrode exhibit significantly stronger signal intensity, with distinct LiF signatures alongside various lithium compounds characteristic of SEI formation. This phenomenon can be ascribed to the lithiophilicity of C-F bonds, which promote Li nucleation. Upon cleavage during the plating process, these bonds generate additional LiF, facilitating the formation of LiF-enriched SEI layers [14]. The F_1s_ spectrum confirmed that fluorine in the F-CP@Ag electrode remains predominantly in a covalent state, while the Ag_3d_ spectrum does not present a metallic Ag peak, suggesting that Ag undergoes a solid-solution alloying process during lithiation, thereby enhancing lithium affinity.

These findings, in conjunction with electrochemical data and post-cycling morphological observations, provide a comprehensive understanding of the mechanisms underlying the superior lithium plating behavior of F-CP@Ag. The formation of a robust, LiF-rich SEI, coupled with enhanced lithium nucleation and reduced energy barriers, contributed to the remarkable cycling stability of F-CP@Ag as a lithium metal current collector.

## 4. Conclusions

In this study, carbon paper, resistant to lithium metal corrosion, was selected as a lithium metal host. By using AgNO_3_ as the silver source and PVDF as the fluorine source, we successfully prepared fluorine-doped, lithiophilic carbon material (F-CP@Ag) with uniformly loaded silver nanoparticles on artificial carbon paper. The amorphous carbon layer doped with uniformly distributed silver nanoparticles and fluorine enhanced the lithium-ion transport and lithiation of the substrate. Ag improved lithium metal wettability and reduced the nucleation energy barrier, while fluorine doping facilitated the formation of a LiF-rich SEI, tolerating lithium metal volume changes. These synergies ensured uniform and stable lithium plating/stripping on the F-CP@Ag electrode.

In half-cells, the F-CP@Ag electrode exhibited stable cycling for 120 cycles at 0.5 mA cm^−2^, 2 mA h cm^−2^. In symmetric cells, stable cycling was maintained for 1400 h at 1 mA cm^−2^, 1 mA h cm^−2^, with a low polarization voltage of 8 mV. Even under harsher conditions of 3 mA cm^−2^, 1 mA h cm^−2^, stable cycling was achieved for over 500 h with a polarization voltage of approximately 38 mV. This study demonstrates that the combination of lithiophilic sites and stable SEI can effectively enhance uniform lithium plating/stripping.

## Data Availability

Data are contained within the article.

## Abbreviations

The following abbreviations are used in this manuscript:

MDPI	Multidisciplinary Digital Publishing Institute
DOAJ	Directory of Open Access Journals
TLA	Three-letter acronym
LD	Linear dichroism
